# Expression of the inhibitory checkpoints LAG-3, TIM-3, and PD-1 in NK cells and T cells in acute myeloid leukemia: preserved expression of LAG-3 is associated with patient survival

**DOI:** 10.1007/s00262-025-04169-y

**Published:** 2025-10-06

**Authors:** Isabel Valhondo, Alejandra Pera, Fakhri Hassouneh, Nelson López-Sejas, Pablo Álvarez-Heredia, Juan M. Bergua, María José Arcos, Ignacio Casas-Avilés, Joaquín Sánchez-García, Josefina Serrano, Carmen Martín, Esther Durán, Corona Alonso, Rafael Solana, Raquel Tarazona

**Affiliations:** 1 Área de Inmunología, Departamento de Fisiología, Universidad de Extremadura, 10003 Cáceres, Spain; 2 Departamento de Biología Celular, Fisiología e Inmunología, Universidad de Córdoba, 14004 Córdoba, Spain; 3 Inmunología y Alergia, Instituto Maimónides de Investigación Biomédica de Córdoba (IMIBIC), 14004 Córdoba, Spain; 4 Present Address: Hospital Nostra Senyora de Meritxell, Avinguda Fiter I Rossell 13, AD700 Escaldes-Engordan, Andorra; 5 Servicio de Hematología y Hemoterapia, Hospital San Pedro de Alcántara, 10003 Cáceres, Spain; 6 Unidad de Gestión Clínica de Hematología, Hospital Universitario Reina Sofía, 14004 Córdoba, Spain; 7 Biología Celular en Hematología, Hipercoagulabilidad. Instituto Maimónides de Investigación Biomédica de Córdoba (IMIBIC), Córdoba, Spain; 8 Departamento de Medicina, Universidad de Córdoba, 14004 Córdoba, Spain; 9 Anatomía y Anatomía Patológica Comparada, Facultad de Veterinaria, Universidad de Extremadura, 10003 Cáceres, Spain; 10 Unidad de Gestión Clínica de Inmunología y Alergología, Hospital Universitario Reina Sofia, 14004 Córdoba, Spain; 11 Instituto Universitario de Biomarcadores de Patologías Metabólicas y Moleculares, Universidad de Extremadura, Cáceres, Spain

**Keywords:** Acute myeloid leukemia, LAG-3, NK cells, PD-1, T and NKT-like cells, TIM-3

## Abstract

Acute myeloid leukemia (AML) is the most common type of leukemia in adults. Despite advances in treatment, the average survival rate after diagnosis is low. AML also alters the patient's immune response further contributing to disease progression. The aim of this study was to analyze the expression of LAG-3, TIM-3 and PD-1 inhibitory checkpoints on NK cells and T cells from newly diagnosed AML patients and its impact in patient survival. NK cells and T cells from AML patients showed a lower expression of TIM-3 compared with healthy donors, whereas no significant differences were observed in the expression of LAG-3. The percentages of cells co-expressing these receptors showed a decrease in the percentage of LAG-3 + TIM-3 + PD-1 − NK and T cells from AML patients in comparison with healthy donors. Remarkably, the survival analysis of AML patients according to the expression of these receptors showed that higher expression of LAG-3 on NK cells and T cells was associated with better survival. In addition, those AML patients showing higher co-expression of LAG-3 and TIM-3 on NK and T cells had better survival (higher than 6 months) than those with lower co-expression of LAG-3 and TIM-3, therefore supporting that the expansion of NK and T cells with lower expression of these checkpoint receptors reflects a dysfunctional state associated with poor prognosis. This study identifies the expression of LAG-3 and TIM-3 checkpoints on NK and T cells as potential biomarkers of AML prognosis, contributing to define those patients that could benefit from checkpoint blockade therapies.

**Supplementary Information:**

The online version contains supplementary material available at 10.1007/s00262-025-04169-y.

## Introduction

Acute myeloid leukemia (AML) is a hematologic malignancy of the myeloid lineage, characterized by clonal expansion of immature blast cells that leads to ineffective hematopoiesis and bone marrow failure. It is the most common blood cancer in adults and has a poor prognosis due to its aggressiveness and high relapse rates. Despite advances in treatment, the prognosis remains very poor, especially in elderly patients, highlighting the need to increase our understanding of the biological and immunological aspects in the generation and progression of AML and in the use of novel immunotherapies for the treatment of AML patients [1].

Immune evasion represents a prominent mechanism involved in cancer progression. Thus, several factors have been identified that contribute to leukemia escape and progression including the presence of dysfunctional immune cells. Different phenotypic and functional alterations of T cells and NK cells have been described in AML patients compared with healthy donors that correlate with AML outcome [2–5]. Altered phenotypes of NKT-like cells, a subset of T cells defined by the expression of CD56, have also been described in AML patients. Unlike the minor population of invariant NKT cells that recognize glycolipids restricted by CD1d, NKT-like cells (CD56 + CD3 +) recognize antigens in a CD1d-independent manner. The percentage of NKT-like cells in peripheral blood increases with age, whereas invariant Vα24 NKT cells decrease with age [6] and its role in cancer surveillance has been highlighted in the last decade. We have previously analyzed the axis DNAM-1/TIGIT/TACTILE in AML patients describing a decrease of the activating receptor DNAM-1 in NK cells, T cells and NKT-like cells [3, 4].

Recently, the impact of immune checkpoint dysregulation on AML overall survival, which predicts disease progression, has contributed to the design of new immunotherapeutic strategies targeting checkpoint blockade for AML patients [7–10]. Checkpoint inhibitors represent a great advance in the treatment of different solid cancers. Although the effect of programmed cell death protein 1 (PD-1) blockade in AML as monotherapy is so far disappointing, it has shown promising results in combination with hypomethylating agents [8, 11]. More studies are necessary to evaluate how PD-1 affects the immune response in patients with AML and select those who may benefit from this treatment [12]. Other inhibitory checkpoints expressed on immune cells are lymphocyte activation gene-3 (LAG-3, also called CD223) and T cell immunoglobulin and mucin domain-3 (TIM-3) [13, 14], considered together with PD-1 as exhaustion markers that contribute to T cell and NK cell dysfunction [15–17]. The identification of novel immune checkpoints and their role in cancer immunosurveillance represents an expanding area of research. The combined expression of PD-1 with other checkpoints including LAG-3 in T lymphocytes from bone marrow can be considered biomarkers of poor outcomes of AML patients [18–20]. High expression of LAG-3 alone or in combination with CTLA4 on T cells subsets identifies a subgroup of AML patients with poor prognosis [21]. The possibility of modulating immune response by checkpoint blockade of LAG-3 or TIM-3 alone or in combination with anti-PD-1 is being analyzed in clinical trials [22, 23].

The aim of this work was to analyze the expression of LAG-3, TIM-3 and PD-1 on peripheral blood lymphocytes (NK cells, CD4 and CD8 T cells and CD3 + CD56 + NKT-like cells), in newly diagnosed AML patients compared to healthy donors and its possible relevance as biomarkers of patient survival. Although in the last decade there has been an increase in our knowledge of the immune response against leukemia, many aspects remain unclarified or even controversial regarding the characterization of immune cells in AML patients. The identification of predictive biomarkers of patient survival and suitability for treatment with checkpoint inhibitors represent ultimate goals. Even though high variability was observed in the expression of these receptors in AML patients, our study shows that LAG-3 expression identifies those AML patients with better survival. In addition, the phenotypic pattern of co-expression (LAG3 + TIM-3 + PD-1 −) could also be related to patient survival, On the contrary, although PD-1 expression alone on NK, T and NKT-like cells did not show any association with patient survival, when it was co-expressed with TIM-3 (LAG3 − TIM-3 + PD-1 +) a negative influence on patient survival was detected.

## Materials and methods

### Patients and healthy donors

Twenty-six patients diagnosed with AML between 2017 and 2019 were included in the study and were followed up until the end of January 2020. The mean age of AML patients was 66.12 ± 18.71 (range 25–90) and 70% were over 65 years old at the time of diagnosis. The majority of patients were males (80.77%). Clinical characteristics of AML patients at the time of diagnosis are summarized in Table 1. Twenty healthy volunteer donors (from now on referred to as healthy donors, HD) were also included in the analysis. The mean age of healthy donors was 59.8 ± 19 (range 18–91), 75% were males and 25% were females. Almost half of the patients received standard intensive induction chemotherapy consisting of cytarabine plus idarubicin alone (n = 6) or in combination with midostaurin (n = 4) or quirzatinib (n = 1). Low-intensity therapy was used in some patients including azacitidine (n = 1), azacitidine plus miniFLUGA (n = 1), Venetoclax (n = 1) and FLUGA (n = 1). One patient was treated with all-trans-retinoic acid (ATRA) and arsenic trioxide (ATO). Four patients died before induction therapy was started and loss of follow-up occur in six patients (Table 1).

**Table 1:**
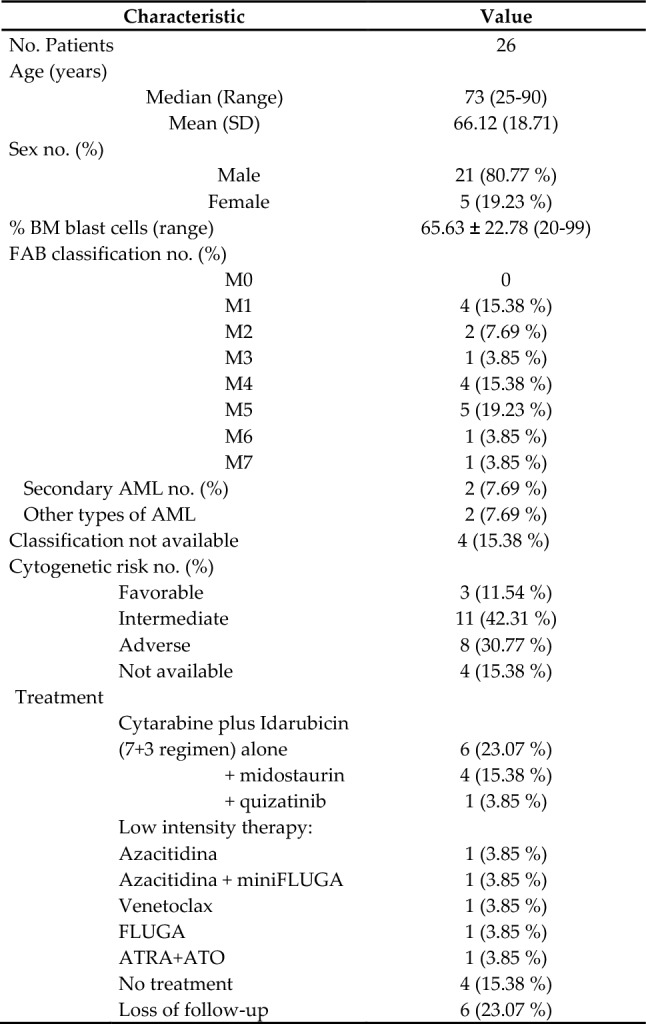
Clinical characteristics of patients with acute myeloid leukemia

The study was approved by the Ethical Committees of the University of Extremadura and Hospital San Pedro de Alcántara (No. 73/2017 to Project SAF2017-87538-R; Cáceres, Spain) and Hospital Reina Sofía (No. 5413 to Project PI21/01125 Córdoba, Spain). The study was conducted in accordance with the Declaration of Helsinki. Peripheral blood samples from AML patients and healthy donors were obtained after informed consent.

Peripheral blood mononuclear cells (PBMCs) were isolated by density gradient centrifugation using Lymphoprep (STEMCELL Technologies, VAN, Canada) and analyzed by flow cytometry as indicated in Sect. "Flow cytometry".

### Antibodies

Flow cytometry analysis of lymphocyte subsets was performed using the following antibodies: anti-CD3 VioGreen (clone: REA613), anti-CD4 PE (clone SK3BD) and anti-CD8 PerCP (clone SK1BD) from BD Biosciences (San José, CA, USA); anti-CD56-PE Vio770, (clone: REA196), anti-CD4 VioBlue, (clone: VIT4), anti-LAG-3 BV421 (clone T47-530BD), anti-TIM-3 BB515 (clone 7D3BD), anti-PD-1 APC (clone MIH4) and anti-CD45 APC-Vio770 (clone REA747) from Miltenyi Biotec (Bergisch Gladbach, Germany).

### Flow cytometry

PBMCs were analyzed for the percentage of the different lymphocyte subsets by multiparametric flow cytometry using MASCQuant flow cytometer (Miltenyi Biotec). Flow cytometry data were analyzed using FlowLogic (Inivai Technologies, Mentone, Victoria, Australia) and FlowJo (v10.0.7, TreeStar, Portland, OR, USA) software. Isotype-matched antibodies and fluorescence minus one (FMO) were used as controls. Lymphocytes were gated according to size and granularity (FSC *vs* SSC) and the expression of CD45. NK cells (CD3− CD56+), NKT-like cells (CD3+ CD56+) and conventional T cells (CD3+ CD56−) were analyzed within the lymphocyte gate. FlowLogic’s Boolean gating options were performed to analyze the co-expression of LAG-3, TIM-3 and PD-1. The gating strategy is shown in Supplementary Figure S1.

UMAP (3.1) and FlowSOM (3.0.18) FlowJo plugins were used to perform UMAP reduction and FlowSOM clustering analysis among lymphocyte cells. For the unsupervised analysis, 1500 events were concatenated within the lymphocyte gate population from each donor. For the clustering analysis, FlowSOM plugin was run using the parameters indicated in each figure and represented as heatmap and density plot.

### Statistical analysis

The statistical analysis was performed using SPSS (version 22.0 statistical package (SPSS, Chicago, IL, USA). Descriptive analysis of variables and normality Shapiro–Wilk tests were used. Mean differences were evaluated by Mann–Whitney U test. Results were considered significant at p value < 0.05. For pie charts comparison we used SPICE permutation analysis [24]. SPICE stands for simplified presentation of incredibly complex evaluations and asks how often the difference observed between the samples represented in pie charts would happen simply by chance. The correlation analysis between the expression of checkpoints on NK and T cells and their correlation with leukemic blasts phenotype was based on the Pearson correlation coefficient and conducted on R-Studio v2022.12.0 + 353. The Kaplan–Meier estimator was used to perform the survival analysis curves. The differences between groups were evaluated using log-rank test upon patient stratification into 2 groups. The Youden's index was used to determine the optimal cutoff point. Where indicated AML patients were categorized as low survivors (< 6 months) or longer survivors (> 6 months) according to the median survival time of AML patients in Spain [25].

## Results

### Expression of LAG-3, TIM-3 and PD-1 in NK cells, T cells and NKT-like cells

**Fig. 1:**
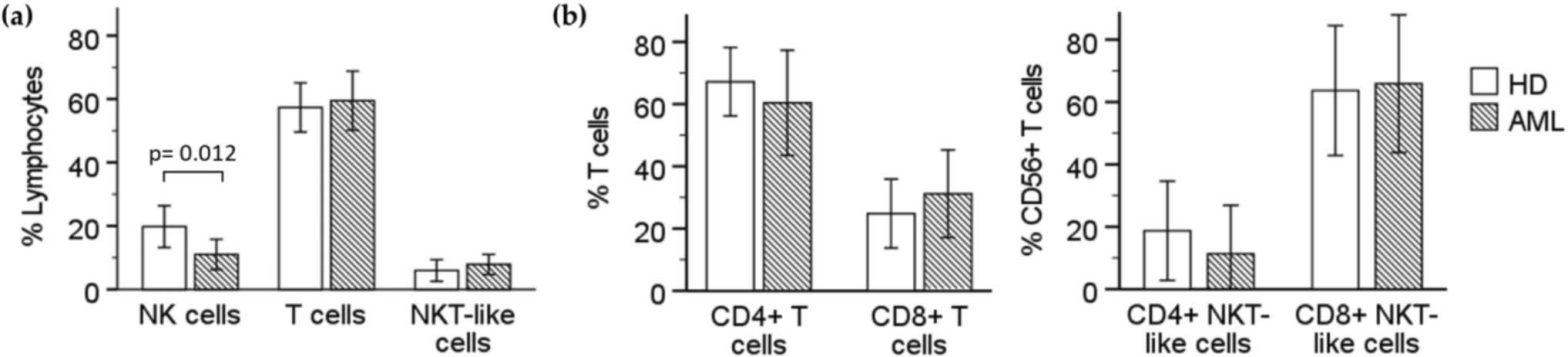
Study of NK cells and T cells in healthy donors and newly diagnosed AML patients. Percentages are referred to the lymphocyte gate. AML: patients with acute myeloid leukemia; HD: healthy donors

AML patients showed a lower percentage of NK cells defined as CD3− CD56+ lymphocytes (mean ± SD; 11.02 ± 12.22) than healthy donors (19.82 ± 14.71). No statistically significant differences were observed in the percentage of conventional T cells (defined as CD3+ CD56− lymphocytes) and NKT-like cells (defined as CD3+ CD56+ lymphocytes) (Fig. 1a). The distribution of T cells and NKT-like cells according to CD4 and CD8 expression did not show statistically significant differences between AML patients and healthy donors. Most NKT-like T cells expressed CD8, both in healthy donors and AML patients (Fig. 1b).

**Fig. 2:**
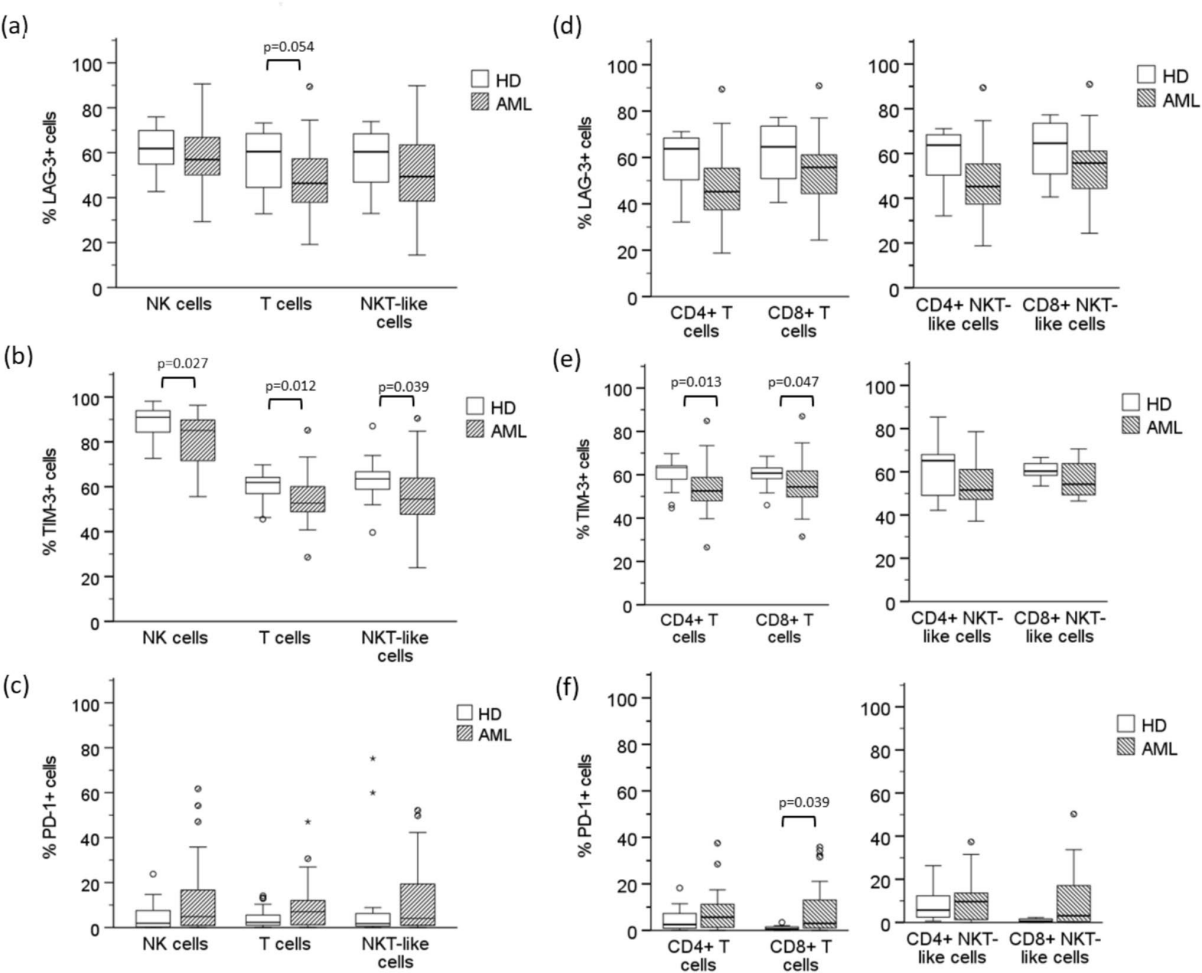
Expression of LAG-3, TIM-3 and PD-1. Percentage of NK cells, conventional T cells and NKT-like cells expressing LAG-3 (**a**; **d**), TIM-3 (**b**; **e**) and PD-1 (**c**; **f**) in healthy donors (n = 20) and newly diagnosed AML patients (n = 26). Vertical lines indicate interquartile ranges from the 25th to the 75th percentile. The horizontal lines represent the median values

The individual analysis of the immune checkpoints LAG-3, TIM-3 and PD-1 on NK cells showed no statistically significant differences in the expression of LAG-3 (Fig. 2a) between AML patients and healthy donors (61.75 ± 10.02 and 59.83 ± 13.66 respectively). TIM-3 (Fig. 2b) was expressed in a lower percentage of NK cells in AML patients (81.93 ± 11.65) compared with healthy donors (89.09 ± 6.94). Although, the expression of PD-1 was higher in AML patients (12.98 ± 17.64) compared with healthy donors (4.85 ± 6.58), the differences were not statistically significant, showing a high variability in the expression of this checkpoint in AML patients (Fig. 2c).

The study of T cells (CD3+ CD56−) showed no statistically significant changes in the expression of LAG-3 (48.58 ± 14.97 in AML and 56.80 ± 14.10 in healthy donors) (Fig. 2a). The percentage of T cells expressing TIM-3 was lower in AML patients (54.18 ± 10.98) compared with healthy donors (59.80 ± 6.76) (Fig. 2b) and no statistically significant differences were observed in the expression of PD-1 (9.55 ± 11.13 in AML and 4.20 ± 4.68 in healthy donors) (Fig. 2c). The analysis according to the expression of CD4 and CD8 coreceptors showed no statistically significant differences in the expression of LAG-3 in CD4 + T cells and CD8 + T cells between AML patients and healthy donors (Fig. 2d). In contrast, TIM-3 was present in a lower percentage of CD4 + T cells and CD8 + T cells in AML patients (Fig. 2e). In AML patients, a higher percentage of CD8 + T cells expressed PD-1 in comparison with healthy donors (Fig. 2f). The analysis of NKT-like cells showed no statistically significant differences in the expression of LAG-3 (51.02 ± 17.17 in AML and 57.01 ± 13.66 in healthy donors) (Fig. 2a), a lower percentage of cells expressing TIM-3 in AML patients (56.85 ± 13.83) compared with healthy donors (62.85 ± 9.44) (Fig. 2b) and no differences in the expression of PD-1 (11.79 ± 15.69 in AML and 9.21 ± 20.29 in healthy donors) (Fig. 2c). The analysis of the expression of these checkpoints in NKT-like cells according to the expression of CD4 and CD8 showed that there are no significant differences between AML patients and healthy donors (Figs. 2d, e and f).

### Co-expression of LAG-3, TIM-3 and PD-1 in NK cells, T cells and NKT-like cells

The study of the differences in the co-expression of these inhibitory receptors was done using SPICE analysis. Pie chart distribution within NK cells was different between AML patients and healthy donors (*p* = 0.0039).

**Fig. 3:**
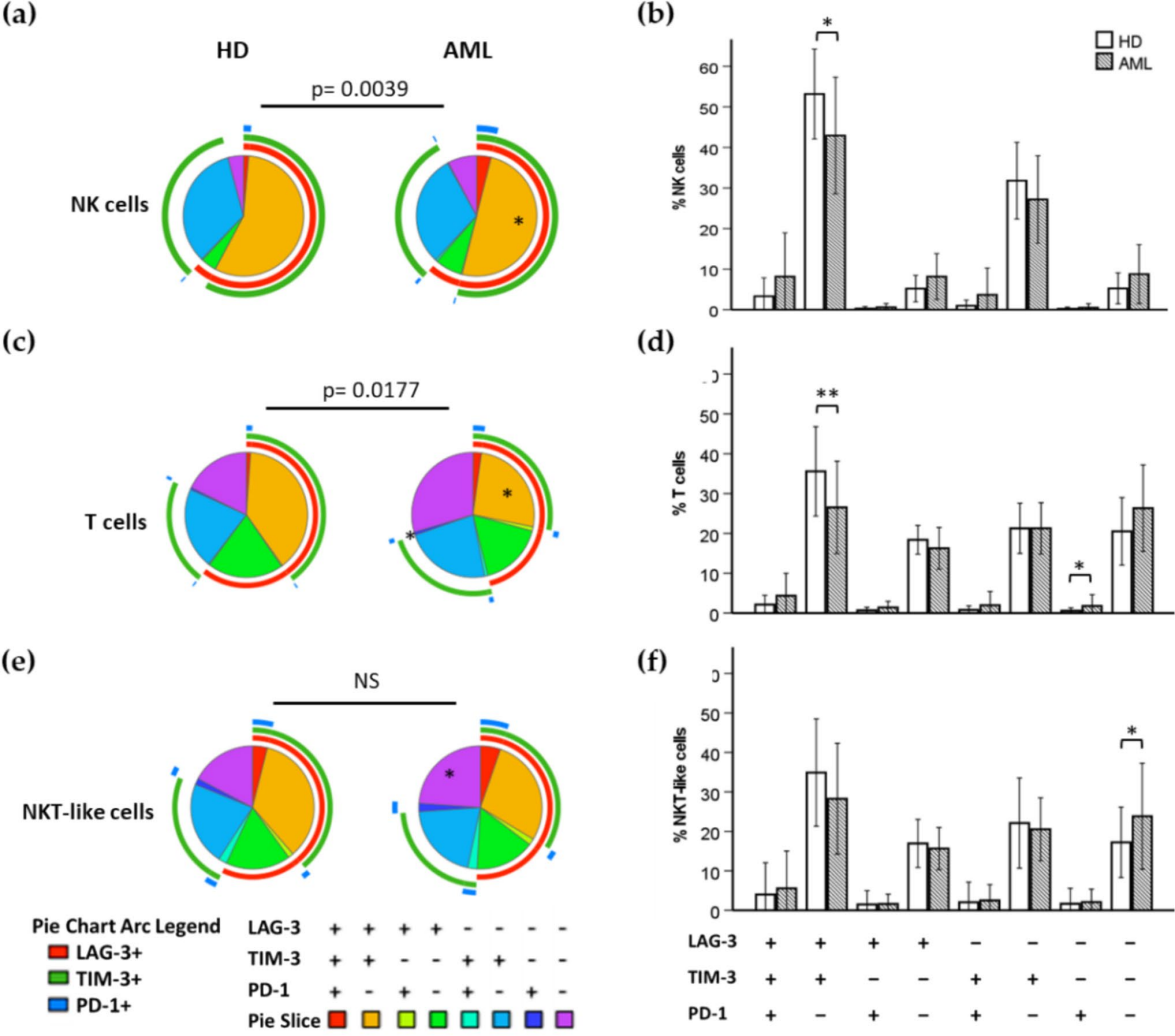
Co-expression patterns of LAG-3, TIM-3 and PD-1 analyzed in NK cells, T cells and NKT-like cells from healthy individuals and AML patients. Eight different subpopulations were observed according to the co-expression of LAG-3, TIM-3 and PD-1 using Boolean gating (**a**, **c**, **e**). Pie charts show LAG-3, TIM-3 and PD-1 co-expression profile in acute myeloid leukemia (AML) patients and healthy donors (HD). Statistical significance was calculated using SPICE permutation tests. Pie arc and pie slice legends are indicated below the pie charts. The asterisk (*) within the slices refers to statistically significant differences between AML patients and HD for the indicated subset. Bar graphs (**b**, **d**, **f**) represent mean ± SD for each subset. *p < 0.05; **p < 0.01.* NS* not significant

In AML patients, NK cells show a lower percentage of LAG-3+ TIM-3+ PD-1− cells in comparison with healthy donors (Fig. 3a, b). SPICE analysis of T cells showed statistically significant differences in the distribution of cell subsets according to LAG-3, TIM-3 and PD-1 expression between AML patients and healthy donors (*p* = 0.0177) (Fig. 3c, d). In contrast, no differences were observed in NKT-like cells (Fig. 3e, f). T cells from AML patients had a decrease in the percentage of LAG-3+ TIM-3+ PD-1− cells and an increased percentage of LAG-3− TIM-3− PD-1+ cells compared with healthy donors. The analysis of the co-expression of LAG-3, TIM-3 and PD-1 in NKT-like cells in AML patients showed a statistically significant increase of the percentage of NKT-like cells that did not express any of these three inhibitory receptors (LAG-3− TIM-3− PD-1−).

The pattern of co-expression of TIM-3, LAG-3 and PD-1 observed in NK cells differs from that found in T cells (*p* < 0.0001) and NKT-like cells (*p* < 0.0001), in both healthy donors and AML patients whereas a similar pattern of co-expression was observed between T and NKT-like cells (Fig. 3 and supplementary Figure S2). TIM-3 is expressed in a higher percentage of NK cells compared with T cells (*p* < 0.001) and with NKT-like cells (*p* < 0.001) in both AML patients and healthy donors. LAG-3 is also found expressed in a higher percentage of NK cells compared with T cells (*p* = 0.011 in both AML patients and healthy donors) and with NKT-like cells (*p* = 0.046 in AML patients and *p* = 0.005 in healthy donors). No differences were observed in the expression of PD-1 among NK cells, T cells and NKT-like cells.

The analyses of LAG-3, TIM-3 and PD-1 co-expression in T cells and NKT-like cells were determined in CD4+ and CD8+ subsets (supplementary Figure S7). Comparison of the profiles obtained using SPICE analysis showed that, in AML patients, both CD4 + and CD8 + T cell profiles differ from those obtained in healthy donors (*p* = 0.0178 and 0.0240 respectively). No statistically significant differences were obtained when NKT-like cells were analyzed (supplementary Figure S7a).

In AML patients, both CD4+ and CD8+ T cells showed a decrease in the percentage of LAG-3+ TIM-3+ PD-1− cells and an increased percentage of LAG-3− TIM-3− PD-1− cells (supplementary Figure S7b). No statistically significant differences were observed between AML patients and healthy donors when CD4+ and CD8+ NKT-like cell subsets were analyzed (supplementary Figure S7b).

**Fig. 4:**
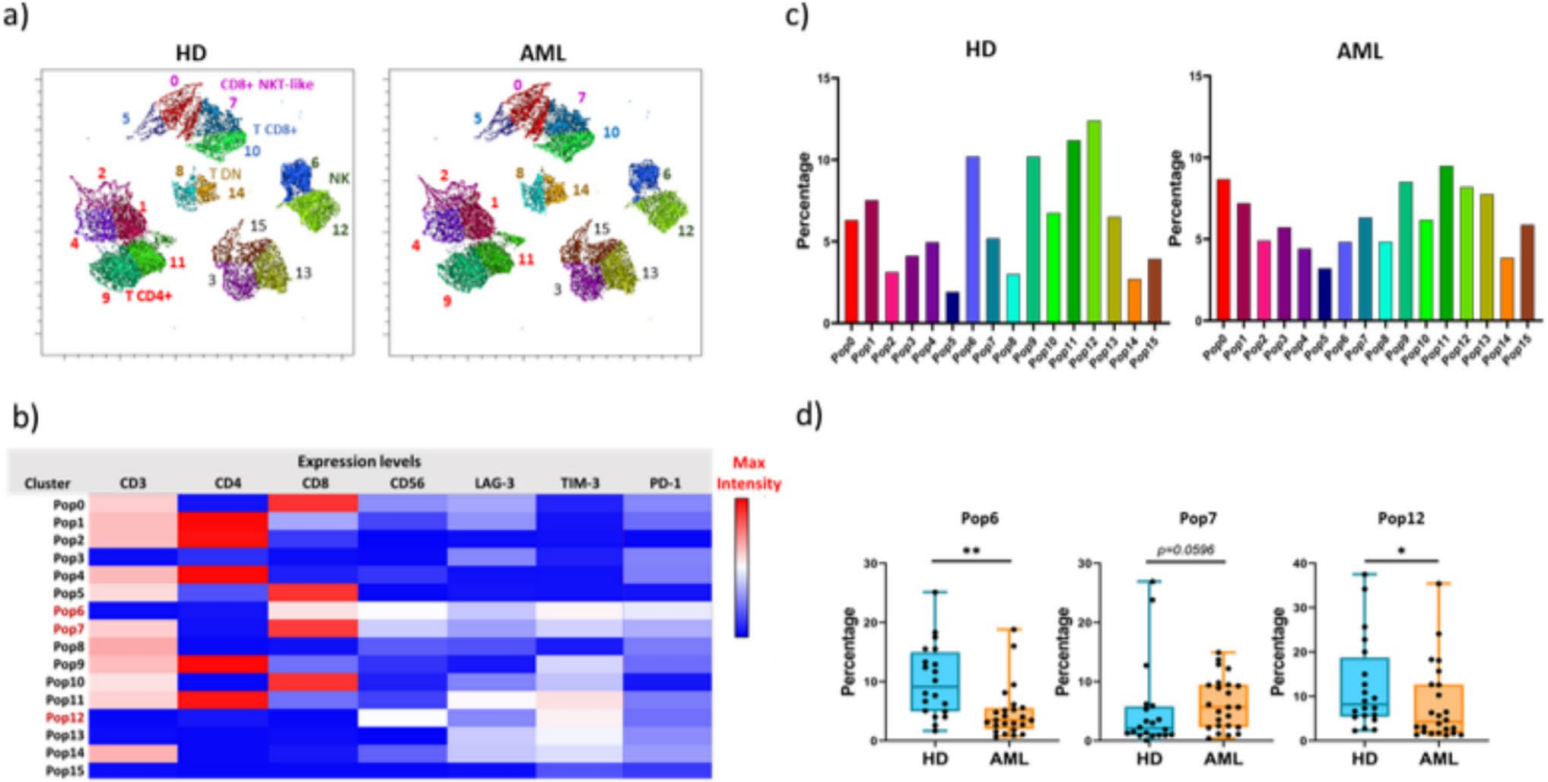
Unsupervised analysis of lymphocyte populations in HD and AML. (**a**) UMAP projection of different lymphocyte populations (Pop) identified by FlowSOM clustering tool. (**b**) Fluorescence intensity for each analyzed marker within each Pop as indicated in the column-scaled z-score (**c**) Bar graphs showing the frequencies of each Pop for each group included in the analysis. (**d**) Boxplot graphs showing the frequencies of Pop6 (NK cells), Pop7 (CD8+ NKT-like cells) and Pop12 (NK cells), for each group. Horizontal bars show the median and whiskers the maximum and minimum values. Each dot represents a donor. Data significance was determined by Mann–Whitney comparison test. *p < 0.05, **p < 0.01, and ***p < 0.001

To identify further differences between AML patients and healthy donors we performed an UMAP reduction and FlowSOM clustering analysis of the samples (Fig. 4). This analysis only revealed statistically significant differences in two NK cells populations (pop 6 and pop 12) that were reduced in AML patients (Pop 6: PD-1+ TIM3+ CD8+ , *p* = 0.0011 and Pop12: TIM3+ , *p* = 0.0463). In T cells, no statistically significant differences were observed but a tendency to increase was observed in CD8+ NKT-like cells (Pop7: PD1+ TIM3+ , *p* = 0.0596).

### Correlation of LAG-3, TIM-3 and PD-1 expression

**Fig. 5:**
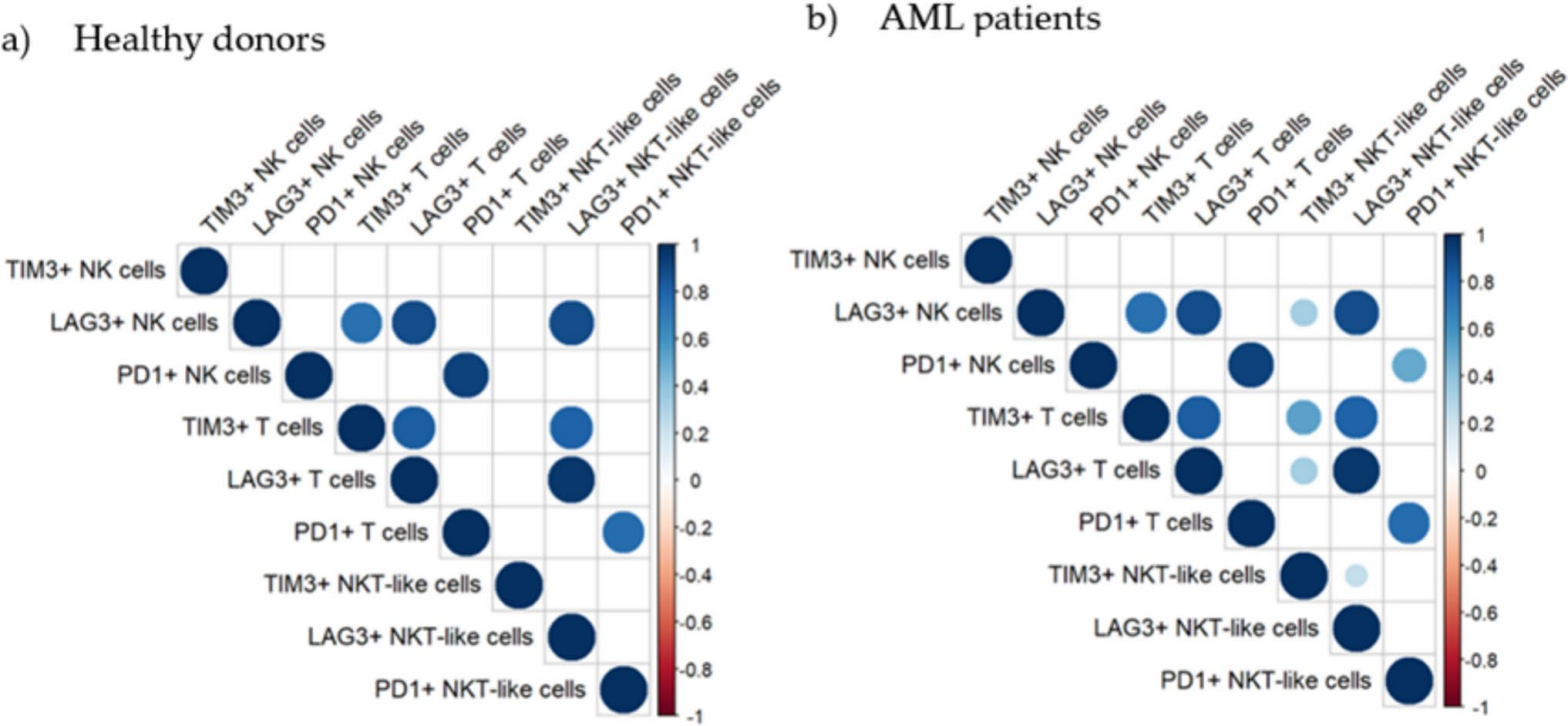
Multiple correlation analysis of TIM-3, LAG-3 and PD-1 expression on NK cells, T cells and NKT-like cells in AML patients. The Pearson correlation coefficient was used to determine multiple correlations. The color and size of the circles in the graph correspond to the obtained r-value; only significant correlations after Bonferroni correction are displayed in the graph

In addition to the analysis of expression level and co-expression patterns, we performed correlation analyses to evaluate whether the expression of TIM-3, LAG-3, and PD-1 in the different lymphocyte subsets is correlated. A similar pattern of positive correlations was found when the expression of TIM-3, LAG-3 and PD-1 was analyzed in healthy donors (Fig. 5a) and AML patients (Fig. 5b). In T cells, a positive correlation was found between the expression of TIM-3 and LAG-3. No correlation in the expression of these receptors was observed when NK cells and NKT-like cells were analyzed. Interestingly, the expression of LAG-3 in T cells positively correlated with its expression in NK cells and NKT-like cells in both healthy donors and AML patients. In patients with AML, TIM-3 expression in T cells positively correlated with its expression in NKT-like cells. Regarding PD-1 expression on T cells, a positive correlation was observed with the percentage of PD-1+ NK cells and PD-1+ NKT-like cells in both healthy donors and AML patients. In addition, in AML patients, PD-1 expression on NK cells also correlated with its expression on NKT-like cells.

### Preserved LAG-3 expression at diagnosis is associated with patient survival

**Fig. 6:**
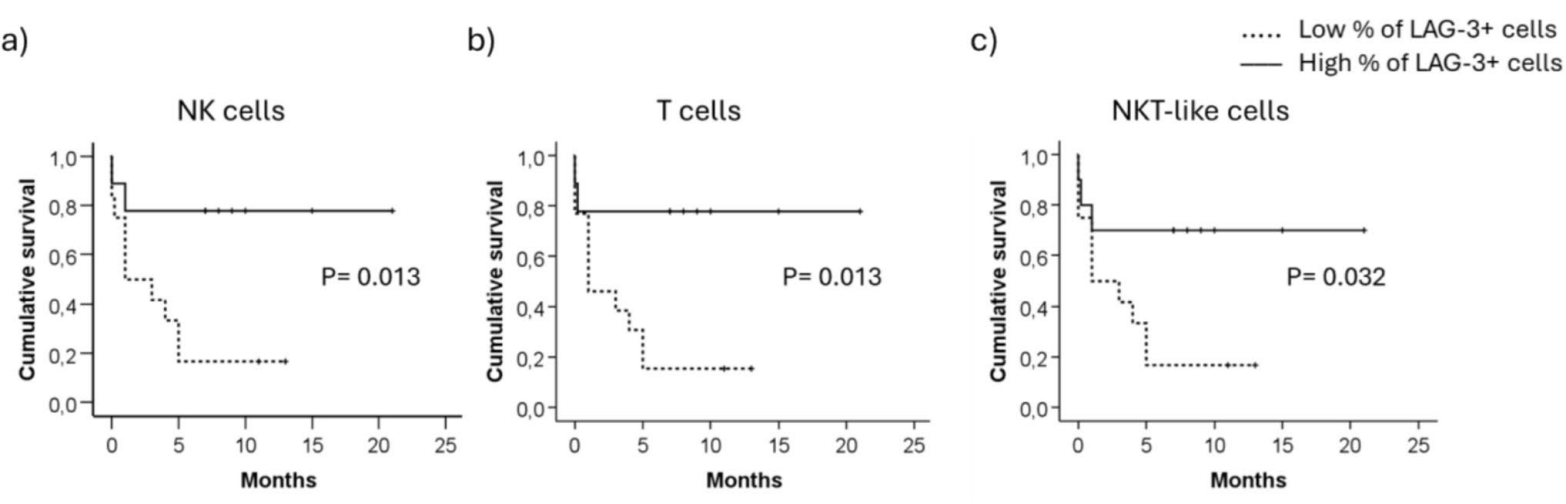
Kaplan Meier survival analysis according to LAG-3 expression. LAG-3 expression was analyzed in NK cells (left panel), T cells (middle panel) and NKT-like cells (right panel). The differences between groups were evaluated using log-rank test upon patient stratification into 2 groups using the Youden's index. Cutoff value was 57% for NK cells, 49% for T cells and 50% for NKT-like cells

To analyze the impact of the expression of these biomarkers in survival, AML patients were classified according to the percentage of cells expressing LAG-3, TIM-3 and PD-1. Interestingly, differences in survival time were found for LAG-3 expression. Thus, those patients with lower percentage of LAG-3+ NK cells (Fig. 6a), LAG-3+ T (Fig. 6b) and LAG-3+ NKT-like cells (Fig. 6c) had inferior survival compared with patients with higher percentages of LAG-3+ cells. In contrast, the expression of TIM-3 and PD-1 on NK cells, T cells and NKT-like cells at the time of diagnoses did not affect patient survival (supplementary Figure S3).

**Fig. 7:**
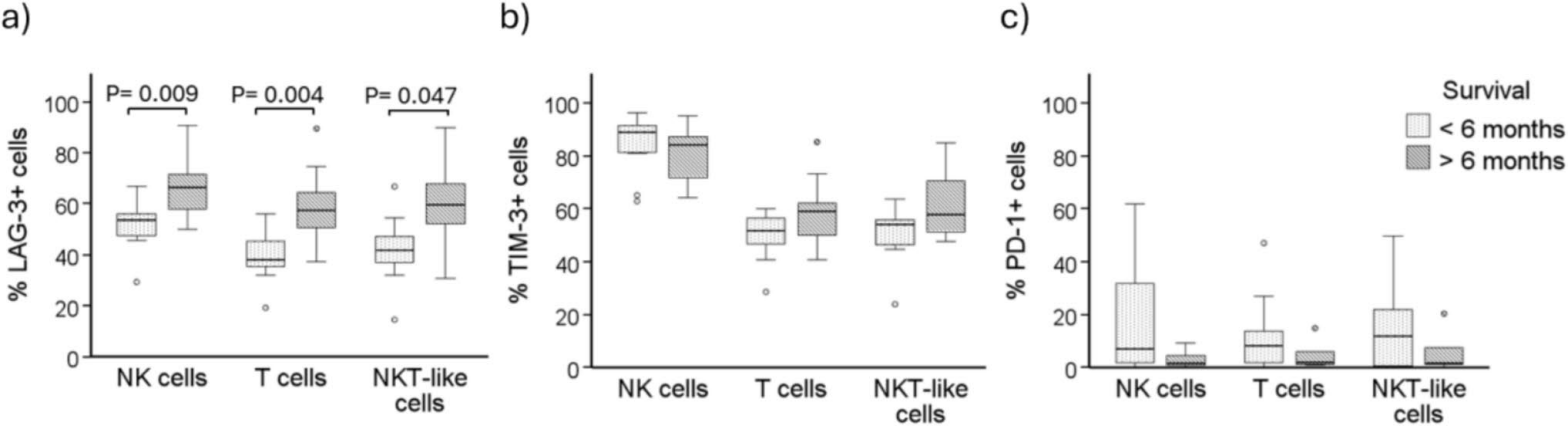
Expression of LAG-3, TIM-3 and PD-1 in AML patients grouped according to survival time. Patients with higher survival (> 6 months) and those with lower survival (< 6 months) were analyzed for the expression of LAG-3 (**a**), TIM-3 (**b**) and PD-1 (**c**) in NK cells, T cells and NKT-like cells

When patients were stratified according to time of survival after diagnosis, those patients that survived less than 6 months had lower percentage of LAG-3+ cells at diagnosis (Fig. 7a). No statistically significant differences were observed when the expressions of TIM-3 (Fig. 7b) and PD-1 (Fig. 7c) were analyzed.

Univariate analysis showed a positive association between the percentage of LAG-3 expression on NK, T and NKT-like cells and survival. When adjusted by patient age at diagnosis, only the percentage of LAG-3+ NK cells and LAG-3+ NKT-like cells maintained their significance as prognostic factors (Table 2).

**Table 2:**
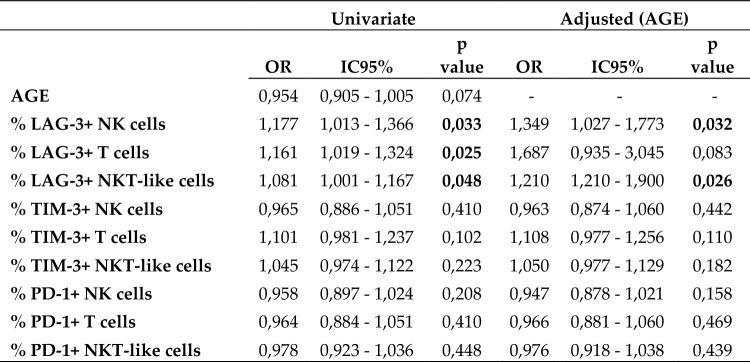
Univariate statistical analysis of factors associated with patient survival

### Analysis of LAG-3, TIM-3 and PD1 co-expression pattern and its association with patient survival

**Fig. 8:**
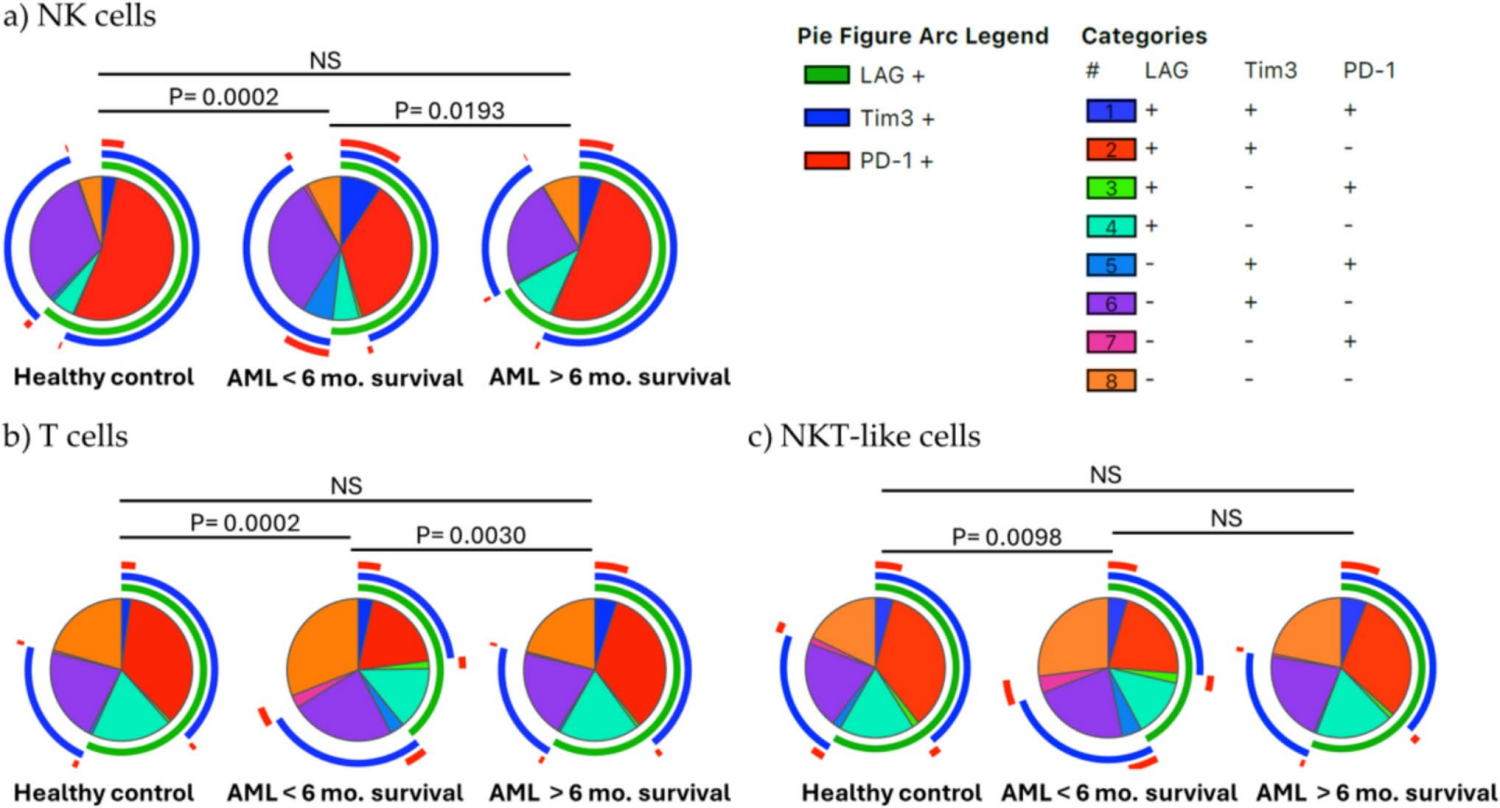
Co-expression patterns of LAG-3, TIM-3 and PD-1 analyzed in NK cells, T cells and NKT-like cells from healthy individuals and AML patients distributed according to survival time. Eight different subpopulations were observed according to the co-expression of LAG-3, TIM-3 and PD-1 using Boolean gating. (**a**) Pie charts show LAG-3, TIM-3 and PD-1 co-expression profile in healthy donors (HD) and acute myeloid leukemia (AML) patients with < or > 6 months of survival. Statistical significance was calculated using SPICE permutation tests. Pie arc and pie slice legends are indicated below the pie charts. *NS* not significant

Analysis of the co-expression of LAG-3, TIM-3 and PD-1 in AML patients and healthy donors showed that those patients with longer survival times had a higher percentage of LAG-3+ TIM-3+ PD-1− NK cells and T cells than those with lower survival and that this percentage was similar to that observed in healthy donors (Fig. 8).

In AML patients, Kaplan–Meier survival analysis according to the co-expression patterns of LAG-3, TIM-3 and PD-1 showed that LAG-3+ TIM-3+ PD-1+ in NK, T and NKT-like cell subsets is higher in patients with lower survival although the differences were not statistically significant (Supplementary Figure S4).

In addition, LAG-3− TIM-3+ PD-1+ NK, T and NKT-like cells (Supplementary Figure S5) were higher in those patients with shorter survival (*p* = 0.037, *p* = 0.097 and *p* = 0.044 respectively), it is interesting to note that this subset, although small, was almost absent in patients with longer survival and in healthy donors. Finally, the larger subset observed in the co-expression patterns corresponded to LAG-3+ TIM-3+ PD-1− cells. The Kaplan–Meier analysis of this subset in NK, T and NKT-like cells showed lower expression in those patients with shorter survival although the differences were not statistically significant (Supplementary Figure S6).

## Discussion

In AML, leukemic blasts and leukemic stem cells develop different immune evasion mechanisms to escape from host immune response, resulting in the progression of leukemia. One of the most recently recognized immune escape mechanisms is the overexpression of inhibitory checkpoints on immune cells that interact with their ligands on leukemic blasts [26, 27]. Immune checkpoint blockade targeting major inhibitory receptors has evolved cancer therapy approaches with good results in other cancers. However, response rates to checkpoint blockade strategies show great variability among patients and differ by cancer type. The effect of antibody blockade of CTLA-4 and PD-1 in AML has been analyzed in different clinical trials with preliminary results showing lower success rates in comparison with those obtained in solid cancer [27, 28].

Recently a role for LAG-3, TIM-3 and PD-1 as markers of T cell exhaustion has been highlighted [17]. Chronic activation of the immune system, as occurs in cancer and in other states of persistent inflammation, leads to increased levels of these inhibitory receptors that correlate with loss of immune effector function [15–17]. In vitro study of cytokine-induced killer cells showed that blockade of KIR, LAG-3, PD-1 and TIM-3 induced a marked increase in AML blast death [29]. Like T cells, NK cell exhaustion has recently been described associated with chronic infections and cancer progression. Exhausted NK cells downregulate the expression of some activating receptors required for target recognition and lysis and upregulate inhibitory receptors. It has been proposed that the expression of LAG-3, TIM-3 and PD-1 on NK cells is associated with dysfunctional exhausted NK cells and that checkpoint blockade may reverse NK cell dysfunction. However, the involvement of these receptors on NK cell function remains controversial [15, 30, 31]. Here, we have analyzed the expression of LAG-3, TIM-3 and PD-1 on NK cells, T cells and NKT-like cells in newly diagnosed AML patients. We found that the expression of these inhibitory checkpoints strongly correlated with each other, in both healthy donors and AML patients, as previously described in patients with other types of cancers [32, 33] suggesting that their role in the tumor microenvironment may affect immunosurveillance and serve as potential therapeutic targets.

Increased LAG-3 expression is considered a T cell exhaustion marker, associated with T cell dysfunction in several human diseases. In our study, no significant differences in the expression of LAG-3 were observed in AML patients compared to healthy donors, probably due to the high heterogeneity observed in the expression of this receptor in patients with AML. Correlation analysis of LAG-3 expressed on lymphocytes and patient cytogenetics did not show statistical significance (data not shown). In our cohort, the low number of patients with favorable cytogenetics limits the statistical power. Multivariate analysis including possible confounding factors such as age, gender or cytogenetic risk did not show statistically significant results (data not shown).

Discrepant results have been described on the association of LAG-3 expression and patient outcome in different types of cancer [33, 34]. Interestingly, we observed that the expression of LAG-3 on peripheral blood NK cells, T cells and NKT-like cells in AML patients is related to survival time, showing that those patients with higher LAG-3 expression had better overall survival compared with those patients with lower expression of LAG-3. Different explanatory possibilities may justify this apparent paradox. In T cells, LAG-3 is stored in lysosomal compartments and translocated to the cell surface after TCR stimulation; otherwise, LAG-3 is degraded in lysosomal compartments [35]. Therefore, in patients with AML, higher LAG-3 expression at diagnosis could indicate greater activation of their immune system.

Our results are consistent with a model where the expression of inhibitory receptors is upregulated upon stimulation to prevent deleterious responses and tissue damage [32]. Accordingly, low expression of inhibitory receptors in lymphocytes from AML patients may reflect a low activation state and consequently reduced immune surveillance. In support of this, it has been described that the expressions of LAG-3, TIM-3 and PD-1 identify antigen-experienced T cells in cancer patients [36]. In addition, since AML is mainly a disease of older adults, we cannot exclude that immunosenescence may also contribute to a state of dysfunctional T cells and NK cells showing a high threshold for activation [10, 37].

Our results are consistent with those described in pediatric patients with Hodgkin lymphoma in which immunohistochemistry analysis showed that those with high density of LAG-3 expression in tumor-infiltrating lymphocytes tended to have better event-free survival compared to patients with lower expression [38]. Furthermore, a meta-analysis study of the prognostic role of LAG-3 expression in cancer patients (breast, ovarian, gastric, lymphoma, non-small cell lung cancer, colorectal, renal cancers and pediatric neuroblastoma) showed an association of LAG-3 expression with a better overall survival in early-stage solid tumors [34].

Altogether, we propose that the expression of LAG-3 on T and NK cells constitutes a marker of immune response against blasts, explaining why those patients with higher percentage of T and NK cells expressing LAG-3 had better survival. Regarding LAG-3 expression, our findings show that in AML patients the preserved expression of this receptor can be associated with longer survival. Thus, LAG-3 blockade may represent a useful strategy to improve the immune response in cancer patients with high LAG-3 expression as it is suggested in a clinical trial in progress (NCT04913922) aiming to study the effect of antibody blockade of LAG-3 and PD-1, in combination with 5-Azacytidine, on survival of patients with relapsed/refractory and elderly AML patients [39].

Our results confirm previous studies showing that TIM-3 expression is higher on NK cells than in T cells, both in healthy donors and in AML patients [40]. We observed a significant decrease in the percentage of NK cells and T cells expressing TIM-3 in AML patients compared with healthy donors. Our results differ from those from Rakova et al. [41] that did not find differences in TIM-3 expression in AML patients in comparison with healthy donors. A high heterogeneity in the expression of TIM-3 was observed in AML patients that may explain these differences. Interestingly, it has been reported that NK cells exposed to cancer cells (glioblastoma and prostate cancer) downregulate TIM-3 expression, which correlates with decreased cytotoxicity and lower interferon gamma production [42]. We have not found a correlation between TIM-3 expression in T and NK cells and patient survival (supplementary Figure S2). Galectin-9, a ligand for TIM-3, showed elevated levels in the serum of patients with leukemia. Thus, TIM-3/Galectin-9 constitutes an autocrine loop that has been implicated in the development of AML [43]. In addition, TIM-3 is also expressed in some cancer cells, including AML stem cells [44] making this axis even more complex. The effect of TIM-3 blockade on cancer cells and its role in immunosurveillance requires further investigation. Rakova et al. described that higher TIM-3 expression is associated with functionally licensed NK cell phenotype and better survival in AML patients [41], further supporting that the lower expression of TIM-3 found in our AML cohort can be correlated with a phenotype of dysfunctional immune cells. Thus, the expression of LAG-3 and TIM-3 on NK and T cells may constitute relevant biomarkers of immune response against AML.

Regarding PD-1 expression, our results agree with those of Tan et al. [45], showing an increased percentage of CD8+ T cells expressing PD-1 in AML patients compared with healthy donors and support the role of PD-1 as a biomarker of T cell exhaustion. Remarkably, in cancer patients the presence of high percentage of T cells co-expressing LAG-3 and PD-1 has been associated with dysfunctionality and resistance to PD-1 blockade-based immunotherapies [46]. Xi et al. described an increase in exhausted TIGIT+ PD-1+ CD8+ T cells in the bone marrow of patients with AML compared with healthy donors. Furthermore, a high percentage of TIGIT+ PD-1+ CD8+ T cells in bone marrow predicted a poor prognosis in AML [47].

Finally, in patients with AML, we observed distinct patterns of co-expression of LAG-3, TIM-3, and PD-1 that are associated with survival time. AML patients with co-expression patterns similar to those observed in healthy donors had greater survival. In contrast, patients with shorter survival times had a lower percentage of LAG-3+ TIM-3+ PD-1− lymphocytes. These results further support that the expression of LAG-3 and TIM-3, in the absence of PD-1, correlates with the activation status of NK cells and T cells. Kaplan–Meier analysis also suggests that higher percentages of LAG-3− TIM-3+ PD-1+ cells are negatively associated with patient survival (supplementary Figure S5). A similar trend is observed when analyzed LAG-3+ TIM-3+ PD-1+ cell subsets (supplementary Figure S4). Functional studies are required to further characterize the activation status and effector function of NK cells and T cells of AML patients according to LAG-3, TIM-3 and PD-1 expression patterns. The effect of antibody-mediated checkpoint blockade of LAG-3 and combination therapy using anti-LAG-3 and anti-PD-1 have been reported using in vitro systems [48] and preclinical models [49, 50]. Increased production of several cytokines including IFN-γ and TNFα has been observed as well as a reduction of tumor volume in those preclinical tumor mouse model, supporting the therapeutic benefit of LAG-3 and PD-1 blockade.

There were some limitations to this study. Firstly, our patient cohort is relatively small limiting the statistical power of the study, and cofounding factors such as cytogenetics, age and gender cannot be excluded. In this sense, the heterogeneity of AML types and treatment options may also be considered as potential limitations of the study. Recently, some PD-1, LAG-3 and TIM-3 polymorphisms have been associated with an increased risk for AML [51–53]. We cannot exclude that these polymorphisms could be responsible for the high variability observed in the expression of these immune checkpoints in NK cells and T cells and may correlate with patient survival. In addition, the expression of these immune checkpoints may vary depending on cell differentiation or maturation status and should be considered in further studies.

In basis of the results described by different authors we hypothesize that LAG-3 expression at levels similar or even higher than in healthy donors imply a state of cells prone to activation, activated or even exhausted, whereas the lower expression of LAG-3 characterizes a state of senescent cells with more limited capacity of activation. Additional studies are necessary to better define the pattern of co-expression of LAG-3, TIM-3, PD-1 and other immune checkpoints on NK and T cells in AML patients and its relevance in their response to checkpoint blockade. Checkpoint blockade of inhibitory receptors, although lowering activation threshold, may not be sufficient to trigger an immune response against leukemic blasts in those patients with a defective senescent immune system. Therefore, the identification of predictive biomarkers of response to checkpoint blockade-based immunotherapies will play an important role in selecting the best therapeutic strategy for each patient.

## Conclusions

In this study, we analyzed the expression of LAG-3, TIM-3 and PD-1 inhibitory receptors in newly diagnosed AML patients. High variability in the expression of these receptors was observed in patients with AML, suggesting that the response to checkpoint blockade may differ in each patient. Our data identify an unexpected direct relationship between LAG-3 expression on immune cells and overall survival.

The identification of novel immune checkpoints and the analysis of their participation in cancer immune escape represent key points for the development of more effective immunotherapies in AML. The observed heterogeneity in the expression of these receptors in AML patients could be related to the different response rates to checkpoint blockade strategies observed in clinical trials.

LAG-3, TIM-3 and PD-1 immune checkpoints are exhaustion markers expressed on NK cells and T cells in a coordinated manner. The individual expression of these checkpoints may not reflect the state of activation or exhaustion of the immune system. Therefore, the analysis of the co-expression of LAG-3, TIM-3 and PD-1 in effector immune cells may represent a better biomarker to establish the activation threshold required for overcoming their inhibitory signaling. Thus, checkpoint blockade would not be effective in a weakened immune system and, conversely, could cause excessive toxicity in an overactive immune system. Further analysis in a major cohort of AML patients is necessary to support the role of these inhibitory receptors in the immune response against leukemia and to elucidate the underlying mechanisms involved in their expression on NK cells and T lymphocytes.

## Supplementary Information

Below is the link to the electronic supplementary material.

Supplementary file1 (PDF 1600 KB)

## Data Availability

The raw data supporting the conclusions of this article will be made available by the authors, without undue reservation.
