# Supplementary material for: Expression of the inhibitory checkpoints LAG-3, TIM-3, and PD-1 in NK cells and T cells in acute myeloid leukemia: preserved expression of LAG-3 is associated with patient survival
Source: Cancer Immunol Immunother. 2025 Oct 6;74(11):325. doi: 10.1007/s00262-025-04169-y (PMC12500513; doi:10.1007/s00262-025-04169-y)
Supplement: Supplementary file 1 — Supplementary file1 (PDF 1600 KB) [file 262_2025_4169_MOESM1_ESM.pdf]

Supplementary material

Valhondo et al. Expression of the inhibitory checkpoints LAG-3, TIM-3, and PD-1 in NK cells and T cells from Acute Myeloid Leukemia patients: Preserved expression of LAG-3 is associated with survival

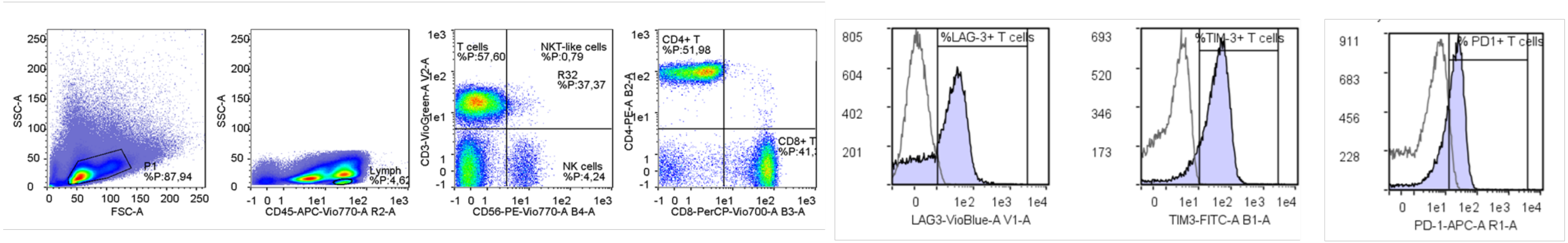

Figure S1. Gating strategy used for the analysis of LAG-3, TIM-3 and PD-1 expression. Lymphocytes were selected according to size and granularity (FSC vs. SSC) and CD45 expression, subsequently NK cells (CD3− CD56+), conventional T cells (CD3+ CD56−) AND NKT-like cells (CD3+ CD56+) were gated by confronting CD56 vs CD3 and followed by the identification of CD4+ and CD8+ T cells. The expression of LAG-3, TIM-3 and PD-1 was determined for each lymphocyte subset. Fluorescence minus one (FMO) and isotype controls were used as controls.

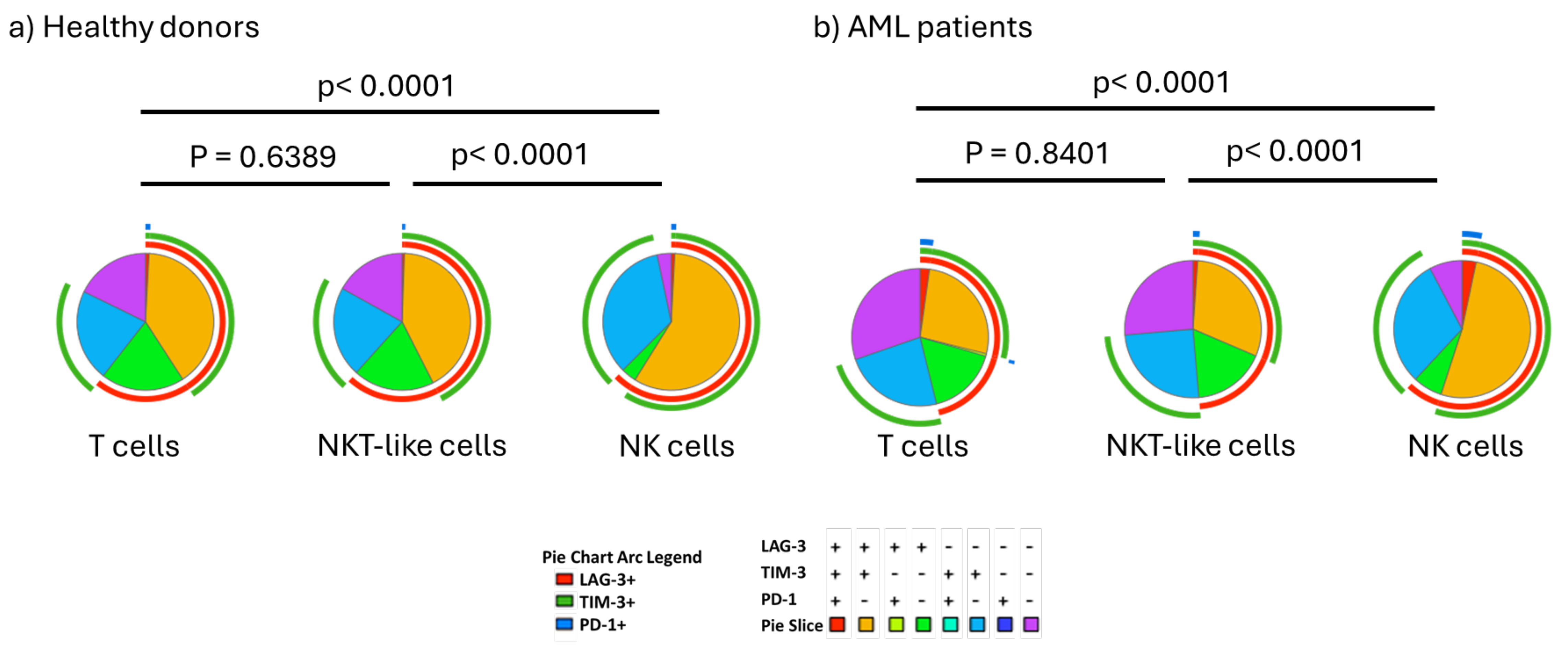

Figure S2. SPICE analysis of LAG-3, TIM-3 and PD-1 co-expression profiles. Pie charts show LAG-3, TIM-3 and PD-1 co-expression profile in different lymphocyte subsets (T cells, NKT-like cells and NK cells) in healthy donors (a) and acute myeloid leukemia (AML) patients (b). Eight different subpopulations were observed according to the co-expression of LAG-3, TIM-3 and PD-1 using Boolean gating. Statistical significance was calculated using SPICE permutation tests. Pie arc and pie slice legends are indicated below the pie charts.

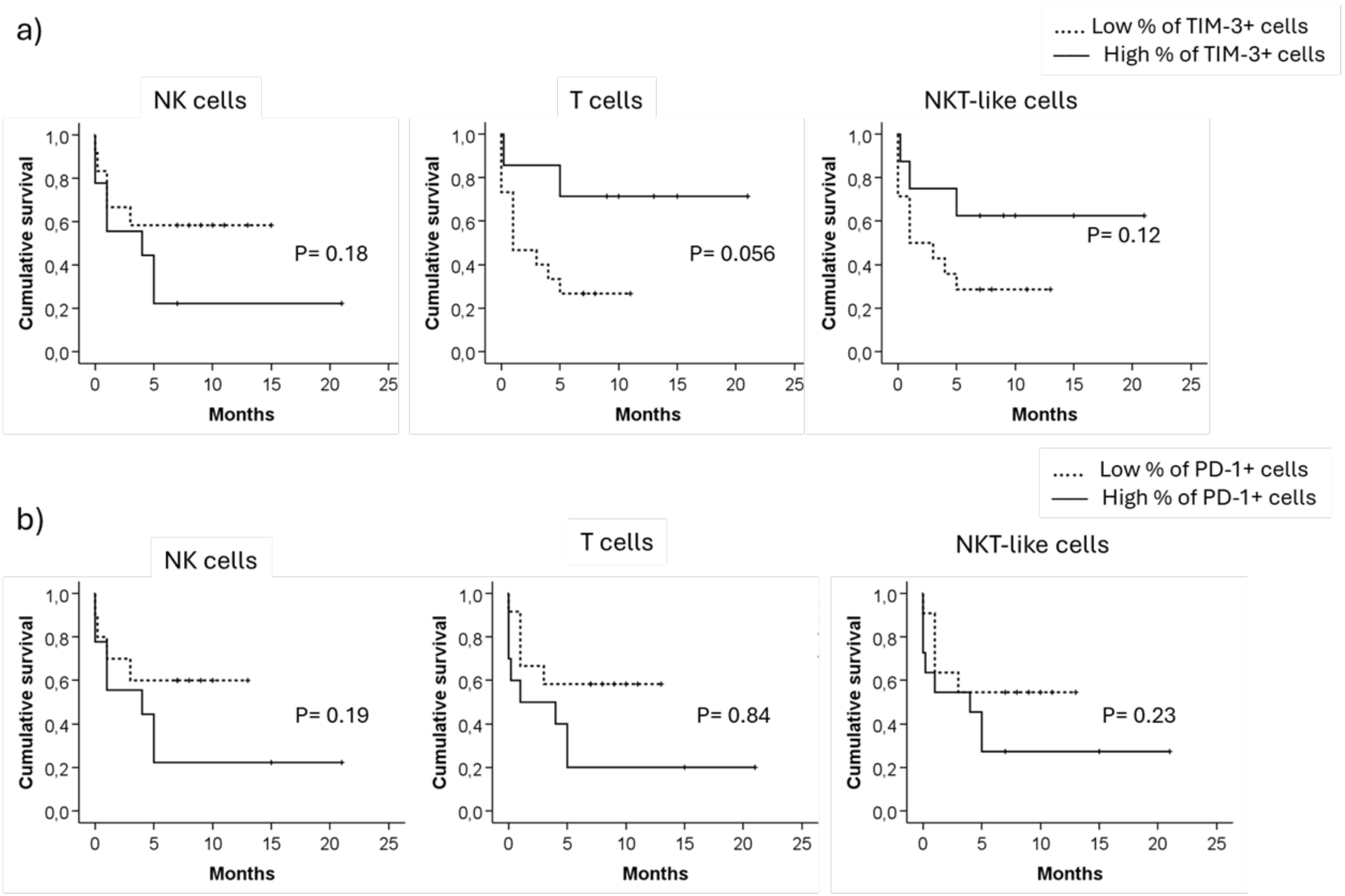

Figure S3. Kaplan Meier survival analysis according to TIM-3 and PD-1 expression. a) TIM-3 expression was analyzed in NK cells (left panel), T cells (middle panel) and NKT-like cells (right panel). The differences between groups were evaluated using log-rank test upon patient stratification into 2 groups using the Youden's index. Cut-off value was 88 for NK cells, 60 for T cells and 57 for NKT-like cells. b) PD-1 expression was analyzed in NK cells (left panel), T cells (middle panel) and NKT-like cells (right panel). Cut-off value was 5 for NK cells, 7 for T cells and NKT-like cells.

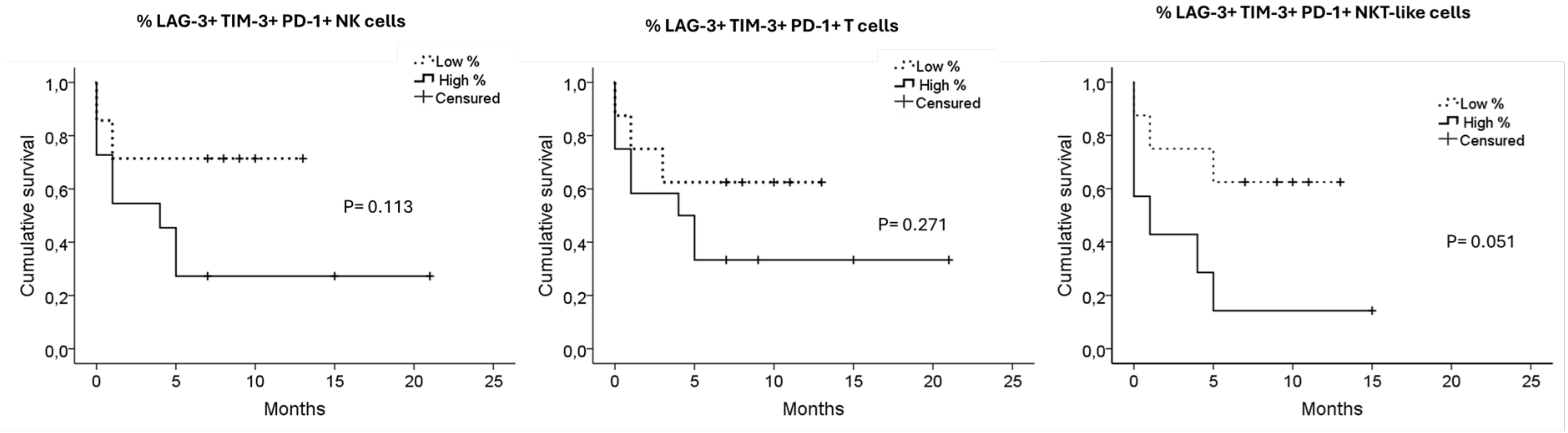

Figure S4. Kaplan Meier survival analysis according to LAG-3, TIM-3 and PD-1 co-expression. The percentage of LAG-3+ TIM-3+ PD-1+ cells was analyzed in NK cells (left panel), T cells (middle panel) and NKT-like cells (right panel). The differences between groups were evaluated using log-rank test upon patient stratification into 2 groups. The median was used as cut-off value (2 for NK cells, 2 for T cells and 4 for NKT-like cells).

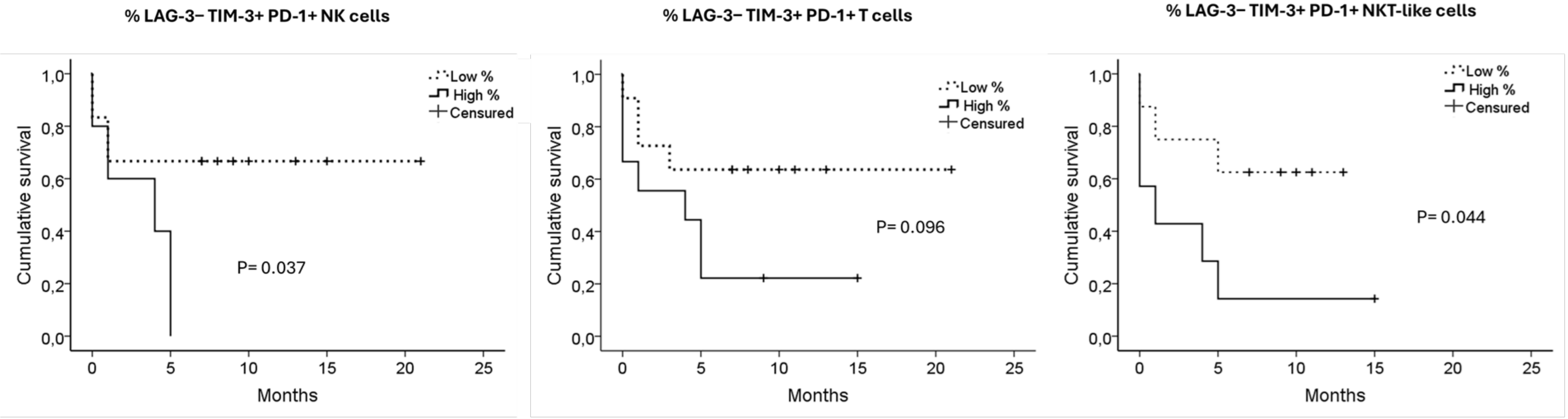

Figure S5. Kaplan Meier survival analysis according to LAG-3, TIM-3 and PD-1 co-expression. The percentage of LAG-3− TIM-3+ PD-1+ cells was analyzed in NK cells (left panel), T cells (middle panel) and NKT-like cells (right panel). The differences between groups were evaluated using log-rank test upon patient stratification into 2 groups. The median was used as cut-off value (4 for NK cells, 1 for T cells and 1.4 for NKT-like cells). This subset is almost indetectable in AML patients with more than six months of survival time and in healthy control donors.

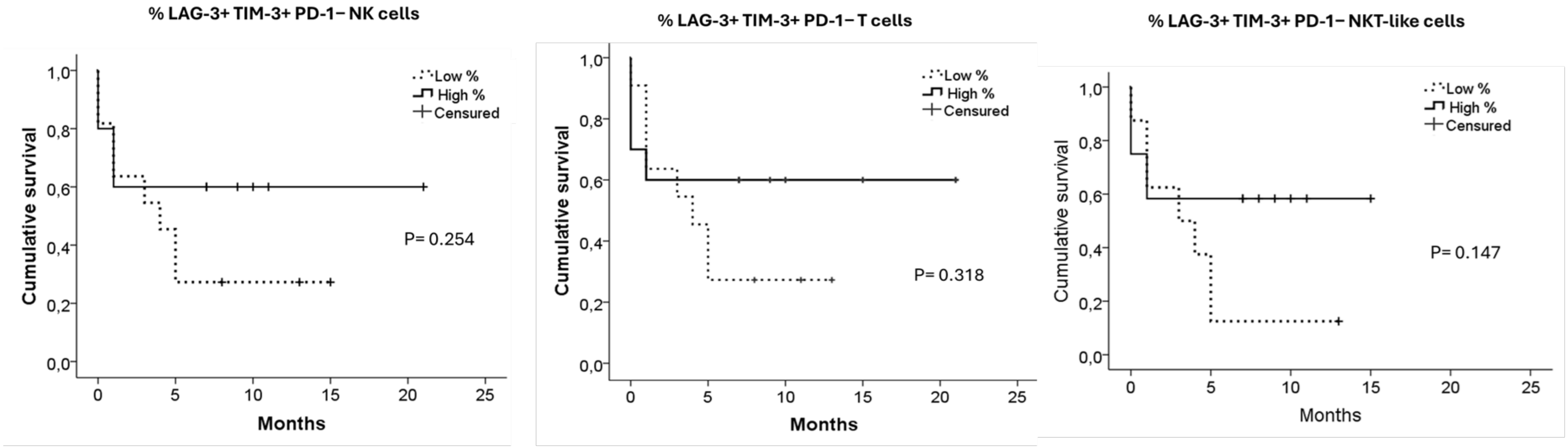

Figure S6. Kaplan Meier survival analysis according to LAG-3, TIM-3 and PD-1 co-expression. The percentage of LAG-3+ TIM-3+ PD-1− cells was analyzed in NK cells (left panel), T cells (middle panel) and NKT-like cells (right panel). The differences between groups were evaluated using log-rank test upon patient stratification into 2 groups. The median was used as cut-off value (45 for NK cells, 25 for T cells and 21 for NKT-like cells).

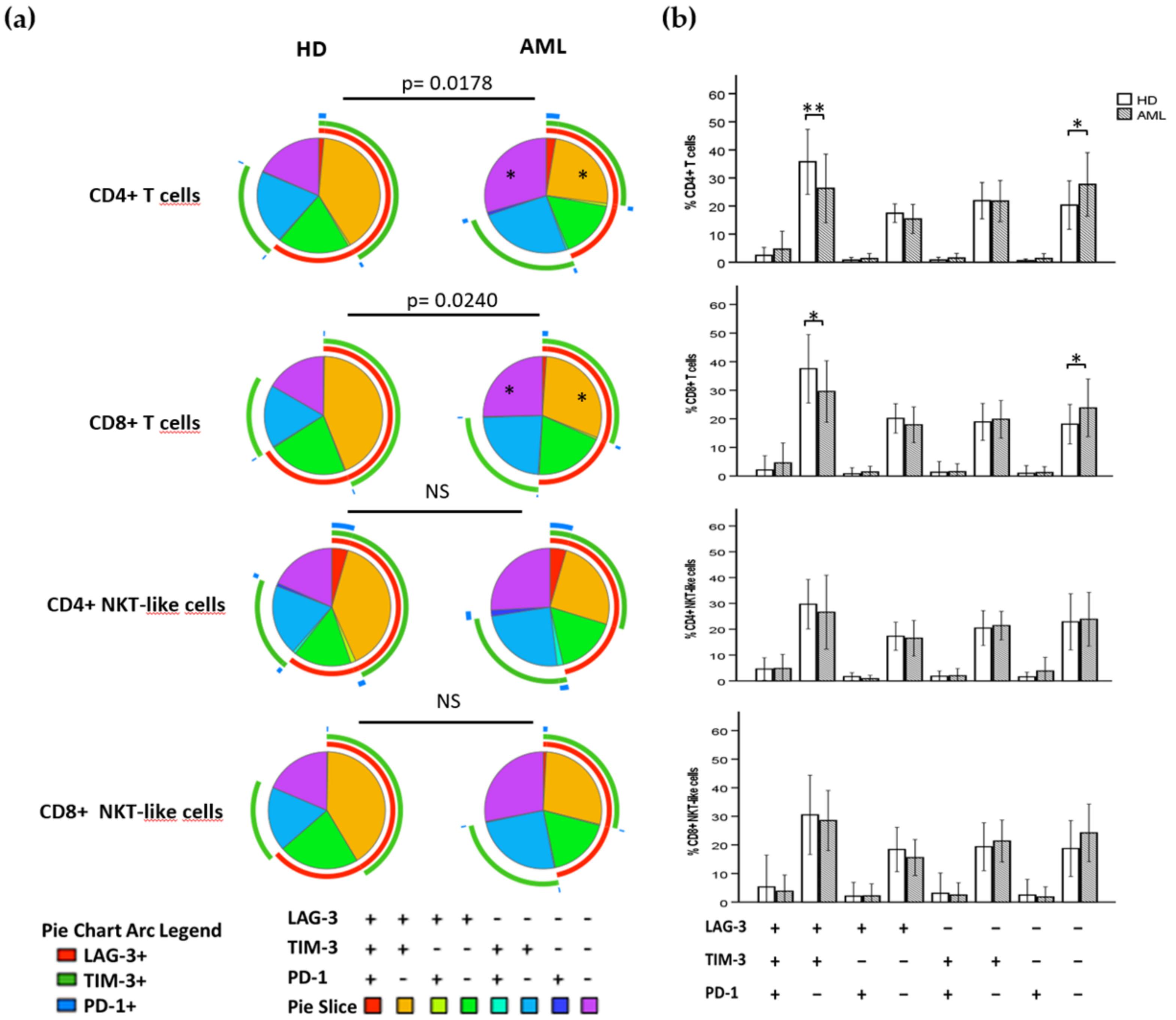

Figure S7. Co-expression patterns of LAG-3, TIM-3 and PD-1 in T and NKT-like cells according to CD4 and CD8 expression. Eight different subpopulations were observed according to the co-expression of LAG-3, TIM-3 and PD-1 using Boolean gating. Pie charts (a) show LAG-3, TIM-3 and PD-1 co-expression profile in acute myeloid leukemia (AML) patients and healthy donors (HD). Statistical significance was calculated using SPICE permutation tests. Pie arc and pie slice legends are indicated below the pie charts. The asterisk (*) within the slices refers to statistically significant differences between AML patients and HD for the indicated subset. Bar graphs (b) represent mean±SD for each subset. *p<0.05; **p<0.01; NS: not significant.
